# Improving the sexual health of women with disabilities in sub-Saharan Africa: a scoping review of published studies

**DOI:** 10.1186/s12978-024-01859-1

**Published:** 2024-08-06

**Authors:** Obasanjo Afolabi Bolarinwa, Clifford Odimegwu, Talent Tapera

**Affiliations:** 1 Demography and Population Studies Programme, Schools of Public Health and Social Sciences, University of the Witwatersrand, Johannesburg, South Africa; 2 Department of Public Health, York St John University, London, UK

**Keywords:** Sexual health, Improving, Women with disabilities, Sub-Saharan Africa, Scoping review

## Abstract

**Background:**

An essential aspect of human well-being is positive sexual health outcomes. However, the issue of adverse sexual health outcomes continues to be a major public health concern, particularly for women with disabilities in sub-Saharan Africa (SSA). Therefore, this current scoping review mapped studies conducted in the last twenty-nine years on the sexual health of women with disabilities from these five dimensions: sexual activity, contraceptive use, sexual autonomy, sexual violence and risky sexual behaviour, whilst seeking to identify the current state of knowledge and address the study gaps in SSA.

**Methods:**

This current scoping review was informed by the methodological framework proposed by Arksey and O’Malley. Exploratory searches were conducted in PubMed, Web of Science, African Journals Online, etc., to identify studies conducted in SSA that focus on sexual activity, contraceptive use, sexual autonomy, sexual violence and risky sexual behaviour among women with disabilities in SSA since the inception of the International Conference on Population and Development in 1994 to 30th of March 2024. This process resulted in the inclusion of seventeen (17) studies.

**Results:**

Of the 1362 identified through various databases, 34 studies were included for the full-text retrieval and screening; only 17 studies met the inclusion criteria. The eligible studies were conducted across six countries in SSA and published between 2008 and 2023. Eight studies used quantitative study type, six utilised qualitative approach, and three employed mixed-methods analysis. Two studies were conducted on sexual activity, ten were conducted on contraceptive use, four were conducted on sexual violence, and one study was conducted on risky sexual behaviour, whilst no study on sexual autonomy met the inclusion criteria.

**Conclusion:**

This review showed that there were few or scarce studies on sexual activity, contraceptive use, sexual autonomy, sexual violence and risky sexual behaviour among women with disabilities in SSA and even where the studies were substantial (contraceptive use), the majority of the studies were conducted in a country. Future studies should consider examining dimensions of sexual health, such as sexual autonomy, sexual activity and risky sexual behaviour of women with disabilities that were not available or were scarce in the literature.

**Supplementary Information:**

The online version contains supplementary material available at 10.1186/s12978-024-01859-1.

## Plain language summary

Sexual health is really important for people's overall well-being, and it includes aspects like how we feel socially, mentally, emotionally, and physically. In sub-Saharan Africa, especially for women with disabilities, sexual health is a big concern. So, this review looked at studies done over the last 29 years about the sexual health of women with disabilities in sub-Saharan Africa. It focused on five areas: sexual activity, contraceptive use, sexual autonomy, sexual violence, and risky sexual behaviour. Databases were searched for relevant studies and found 17 that fit the set criteria. These studies were from six countries in sub-Saharan Africa and were published between 2008 and 2023. Most of the studies were about contraceptive use, with fewer focusing on sexual activity, sexual violence, and risky behaviour. There weren't any eligible studies on sexual autonomy. The review concluded that there's not enough research on these sexual health dimensions among women with disabilities in sub-Saharan Africa, especially on sexual autonomy, and future studies should explore this further.

**Supplementary Information:**

The online version contains supplementary material available at 10.1186/s12978-024-01859-1.

## Background

An essential aspect of human well-being is sexual health, which has social, mental, emotional, and physical aspects [1, 2]. The issue of sexual health continues to be a major public health concern, particularly for women with disabilities in sub-Saharan Africa (SSA). Several studies have reported that achieving optimum sexual health and autonomy is often hindered by a myriad of barriers, including societal stigma, discrimination, and systemic neglect [3–5]. Women with disabilities still face numerous obstacles that obstruct the improvement of their sexual health despite the increased awareness of the significance of sexual health and rights [6]. It is imperative to comprehend and tackle these obstacles to advance gender parity, social justice, and inclusive growth within the region.

Research and advocacy focused on meeting the needs of women with disabilities globally in terms of their sexual health have gained significant traction in recent years [7, 8]. However, much of this attention has been concentrated in high-income countries, leaving behind the unique experiences and concerns of women with disabilities in SSA [9–12]. In the studies conducted by Rugoho and Maphose [13] and Ganle et al. [14] argued that the intersectionality of gender and disability compounds the obstacles faced by women in SSA, where patriarchal norms and cultural taboos further restrict their sexual agency and access to essential healthcare services. This oversight is particularly concerning given the intersecting layers of discrimination and marginalisation faced by women with disabilities in this region, including barriers to healthcare access, limited educational opportunities, and entrenched social stigma [15, 16]. Limited research and scarce data exacerbate the invisibility of this issue, hindering efforts to develop targeted interventions and policies that address the specific needs of women with disabilities.

This current scoping review could serve as a crucial starting point for discussions aimed at improving the sexual health of women with disabilities in SSA. Similar studies conducted in Sweden and Australia led to policy roundtable discussions with stakeholders and policymakers. These discussions resulted in the launch of sexual health initiatives that helped bridge gaps in sexual health services and promoted strong community ties and inclusiveness for women with disabilities in those countries [17, 18].

A scoping review offers a comprehensive approach to mapping the existing literature, identifying gaps, and synthesising evidence on a particular topic [19]. This study presents a scoping review of the sexual health of women with disabilities in SSA. By systematically examining the available literature, the research aims to highlight the gaps in knowledge needed to improve the sexual health of these women since the International Conference on Population and Development (ICPD) in Cairo, Egypt, in 1994, which initiated the concept of sexual and reproductive health and rights (SRHR) [20]. This type of review has been used in many latent studies on improving other specific populations’ sexual health and well-being [21, 22].

The study seeks to accomplish these objectives by presenting a thorough synopsis of the current state of research in relation to sexual health dimensions being considered. Moreover, the study aims to identify areas where evidence is limited, highlighting gaps in knowledge that warrant further investigations or examination with the purpose of improving the sexual health outcomes of women with disabilities in SSA whilst appraising the current state of knowledge to ensure that no one is left behind [23]. It's also pertinent to know that many international agendas, goals and targets might remain unachievable or challenging if the adverse sexual health of women with disabilities is not adequately addressed [24–26].

The study will review and consider a range of sexual health research dimensions that determine the sexual health outcomes of women with disabilities in SSA through sexual activity, sexual autonomy, contraception use, risky sexual behaviour and sexual violence. Through a comprehensive examination of the existing literature, this scoping review seeks to contribute to a deeper understanding of the sexual health current and future research needs for women with disabilities in SSA.

By highlighting neglected gaps and areas that require further investigation, this scoping review aims to catalyse efforts towards the development of inclusive and rights-based approaches to sexual health for all women, regardless of disability status and ultimately achieving sustainable development goals targets 3.7 and 5.6, which, respectively aim to ensure universal access to sexual and reproductive health care services and to achieve gender equality and empower all women and girls [24]. Therefore, the research question for this review is: What is the volume and coverage of current studies on improving the sexual health of women with disabilities in SSA, including aspects such as sexual activity, contraceptive use, sexual autonomy, sexual violence, and risky sexual behaviour?

## Methods

Our scoping review design was guided by the methodological frameworks proposed by Arksey and O’Malley [27]. The two objectives of conducting a scoping review, as outlined by Arksey and O’Malley [27], were achieved by providing a comprehensive summary of existing research on sexual health with a focus on sexual health operationalised from the dimensions of sexual activity, contraceptive use, sexual autonomy, sexual violence and risky sexual behaviour of women with disabilities in SSA also by identifying any gaps present in the current literature. A protocol to guide the scoping review process was developed using the format prescribed by the International Prospective Register of Systematic Reviews (PROSPERO) [28]. However, we did not register the protocol due to PROSPERO's current exclusion of scoping review protocols for registration. We report the process and findings of the scoping review in accordance with the established Preferred Reporting Items for Systematic Reviews and Meta-Analyses (PRISMA) reporting guidelines [29].

### Literature search strategy

Before conducting the full searches for this scoping review, we conducted a preliminary exploratory search of the literature on improving the sexual health of women with disabilities with a focus on sexual activity, contraceptive use, sexual autonomy, sexual violence and risky sexual behaviour in SSA. Searches were performed in Cochrane Library and Joanna Briggs Institute (JBI) databases. Our search revealed that no reviews of this nature had been previously conducted nor were ongoing. However, JBI and Cochrane Library were not considered in the final databases considered. Additionally, exploratory searches were conducted on two general social and behavioural science databases, PubMed and Web of Science, to ensure a comprehensive search. The exploratory review also informed the development of the keyword list for our search strategy, helped refine the research question, and aided in the development of inclusion criteria. More information about the development of the keyword list is available in supplementary appendix I.

We conducted the search using all identified Medical Subject Headings (MeSH) terms found during the exploratory search (Supplementary appendix II). To ensure the inclusion of all relevant studies, particularly those published in African journals, we searched a variety of databases, including PubMed, Web of Science, African Journals Online, JSTOR, and PsycInfo. Additionally, we searched non-database websites such as Google Scholar and Guttmacher to complement or identify any missing study from those included in the databases. Finally, we examined the reference lists of all review studies identified in the search to identify additional relevant literature/eligible study.

### Inclusion and exclusion criteria

#### Population

We included studies conducted among women with any form of disability between the ages of 15 and above. This limits the review to studies conducted on sexual health with a focus on sexual activity, contraceptive use, sexual autonomy, sexual violence and risky sexual behaviour among women with disabilities. We excluded studies that exclusively focus on young girls with disability and studies that compared women with disabilities with women without disability. We excluded studies focusing solely on young girls with disabilities to maintain the focus on adult women. Additionally, studies comparing women with disabilities to those without were excluded to avoid comparative bias and concentrate on the unique challenges faced by women aged 15–49 with disabilities. This ensures a clearer understanding of their specific sexual health needs.

#### Concept

Studies that discussed or assessed sexual health dimensions such as sexual activity, contraceptive use, sexual autonomy, sexual violence and risky sexual behaviour [30, 31] were included in this study, whilst other studies without the stated focus were excluded. The definition of sexual health dimensions considered are in Table 1 below;

**Table 1:** Key terms for measuring sexual health

Term	Dimensions
Sexual Health	Sexual activity, contraceptive use, sexual autonomy, sexual violence and risky sexual behaviour

#### Context

This scoping review covers studies conducted in SSA from January 1st 1995 to 30th March 2024. The 1995 start date was selected as it coincides with the ICPD held in Cairo, Egypt, in September 1994, which marked the inception of the doctrine of SRHR [20].

#### Types of studies

The study types included in this review were qualitative, quantitative, and mixed-methods. Only peer-reviewed studies published in English with the listed study types were considered. Technical reports, thesis, research protocols, book reviews, quasi-experiments, randomised control trials, conference proceedings, commentaries, blog posts and other kinds of grey were excluded. This approach helps maintain a high standard of evidence quality and relevance, focusing on established findings rather than preliminary or non-peer-reviewed work [27].

#### Eligible studies selection process

We employed EndNote X9 (Clarivate), a citation manager, to remove duplicate search results. Subsequently, we utilised the online systematic review software Covidence to conduct screening and data extraction for the remaining search results [32]. Two reviewers independently evaluated each search result. Initially, all titles and abstracts were screened for relevance. Following this, the full texts of all pertinent results were assessed against the inclusion and exclusion criteria. Disagreements at each stage were resolved through discussion until a consensus was reached. In cases where agreement could not be reached, a third reviewer (an independent reviewer) facilitated resolution. This process resulted in the inclusion of 17 studies.

**Fig. 1:**
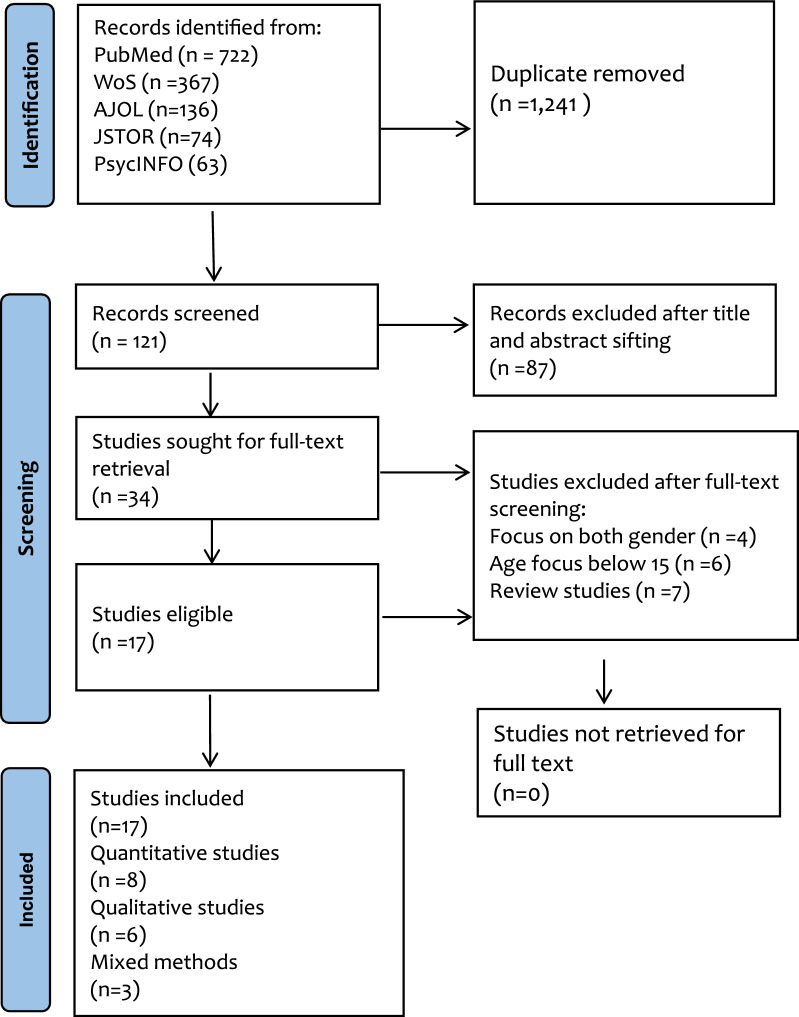
PRISMA 2020 Flow diagram [33]

The elimination stages, the number of results eliminated at each stage, and the reasons for exclusion using the PRISMA flow diagram were displayed in Fig. 1 [29].

#### Data extraction and charting

The included eligible articles were extracted independently from covidence using a standardised form in Microsoft Excel. Specifically, the following information was extracted: the first author's surname and year of publication, study country, and sexual health dimensions, including sexual activity, contraceptive use, sexual autonomy, sexual violence and risky sexual behaviour. Two authors conducted the data extraction, and the full information for all articles included in the review is available in Table 2.

**Table 2:** Study characteristics and key findings

S/No.	First authors surname (year)	Country	Study type	Sexual health of women with disabilities					Key findings
				Sexual activity	Contraception use	Sexual autonomy	Sexual violence	Risky sexual behaviour	
1	Opoku (2016) [47]	Ghana	Qualitative				X		Sexual violence exacerbated by poverty
2	Tessema (2015) [34]	Ethiopia	Quantitative		X				24.3% of unmet need for family planning
3	Ayiga (2016) [45]	Uganda	Quantitative		X				26.1 had utilised contraceptive
4	Beyene (2019) [35]	Ethiopia	Quantitative		X				18% had ever used contraceptive
5	Mesfin (2020) [36]	Ethiopia	Quantitative		X				33.7% had ever used contraceptive
6	Tsegay (2017) [37]	Ethiopia	Quantitative		X				27.2% ever used contraceptive. Injectable (93.2%), oral pills (6.1%) and implants (0.7%)
7	Yimer (2019) [38]	Ethiopia	Mixed-methods		X				High awareness (97.2%) of family planning methods but low comprehensive knowledge of modern contraceptive methods
8	Alem (2021) [39]	Ethiopia	Quantitative		X				Unmet need for family planning among rural women at 24.08%
9	Makau (2021) [49]	Kenya	Mixed-methods		X				Low family planning sutilisation in Kajiado County due to negative attitude
10	Van der Heijden (2019) [44]	South Africa	Qualitative	x					Disability stigma may impede the achievement of normative womanhood and sexual relationships
11	Tenaw (2023) [40]	Ethiopia	Quantitative		X				27.3% were using contraceptives, with 48.5% using implants
12	Tenaw (2023)[41]	Ethiopia	Quantitative				X		A high prevalence of sexual violence (59.8%) is associated with residing in an urban setting
13	Kvam (2008) [50]	Malawi	Qualitative				X		Childhood sexual abuse was not reported, but in adulthood, many experienced what they defined as sexual abuse
14	Birungi (2023) [46]	Uganda	Qualitative					X	Risky sexual behaviour is prevalent and often associated with unwanted pregnancies and sexual encounters with strangers
15	Hanass-Hancock (2009) [42]	South Africa	Qualitative	X					Sexual abuse and exploitation are emerging as significant threats to HIV/AIDS prevention
16	Nketsia (2022) [48]	Ghana	Mixed-methods		X				Deaf women have limited knowledge of contraceptive methods because of barriers to accessing services
17	Phasha (2012) [43]	South Africa	Qualitative				X		Learners with intellectual disabilities are vulnerable to school-based sexual violence

x = available

## Results

The initial search conducted by the authors had 1362 studies, and 1241 duplicates were removed as duplicates. One hundred twenty-one (121) were included for title and abstract sifting, and 87 studies were removed. Thirty-four studies (34) were included for the full-text retrieval and screening, and only 17 studies were included as eligible studies, whilst 17 studies were excluded due to not meeting the inclusion criteria (Fig. 1).

### Geographical locations covered

**Fig. 2:**
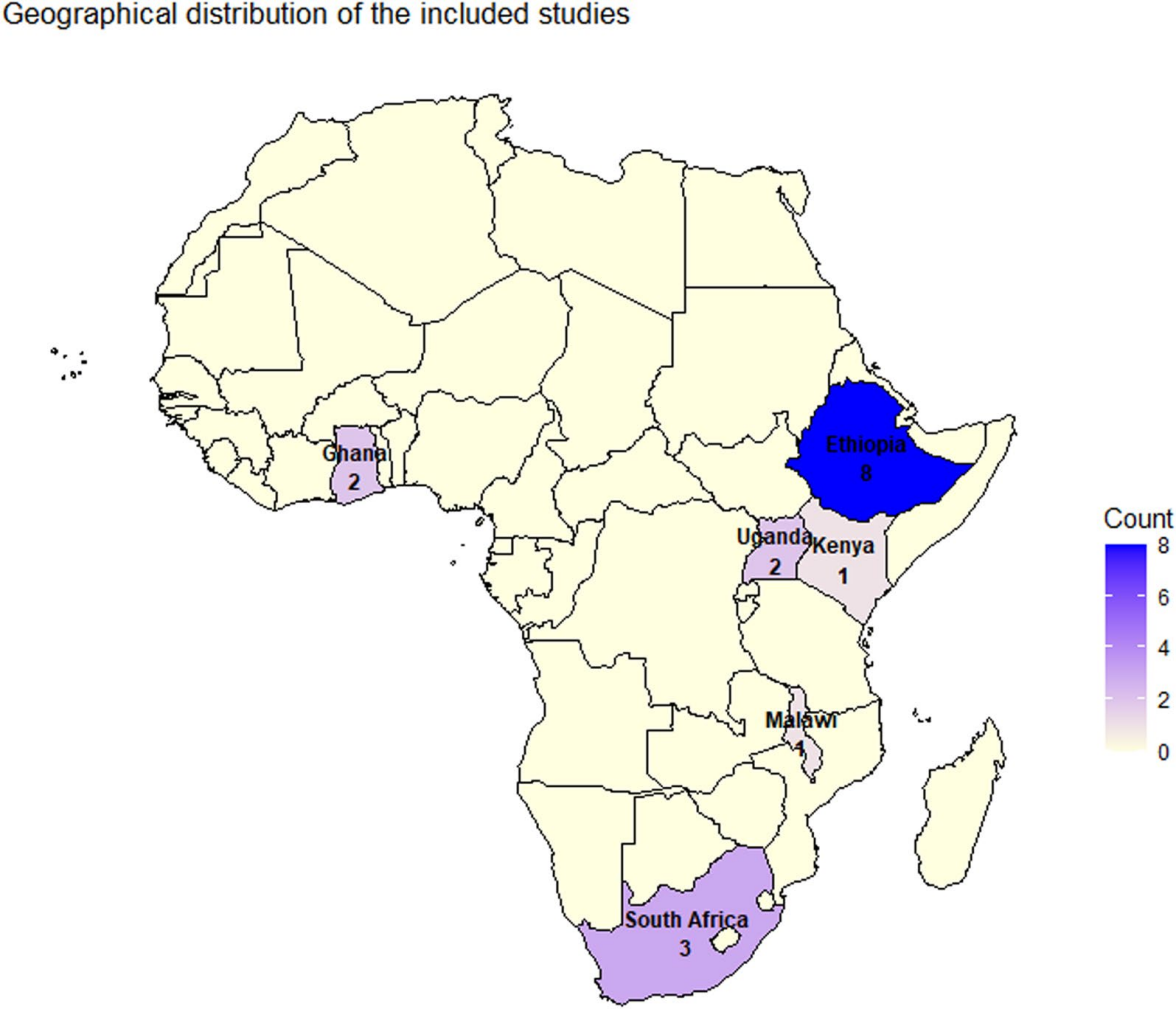
Geographical distribution of studies included

The seventeen (17) included studies were conducted across 6 countries in SSA and published between 2008 and 2023. Eight out of the eligible studies were conducted in Ethiopia [34–41], three from South Africa [42–44], two from Uganda [45, 46], two from Ghana [47, 48] and one each from Kenya [49] and Malawi [50] (Table 2; Fig. 2).

### Study type

**Fig. 3:**
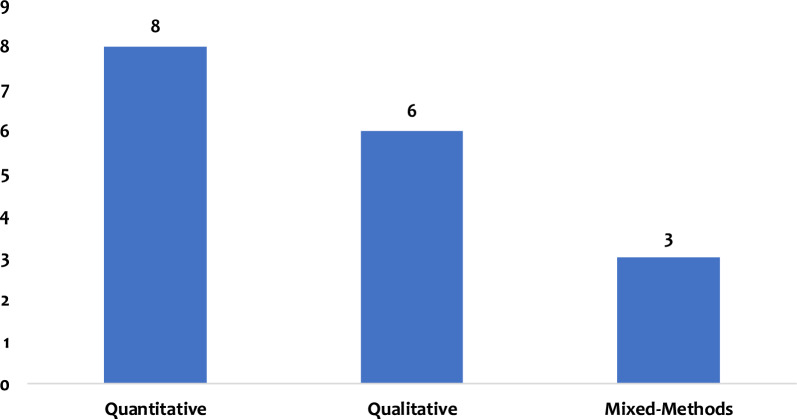
Study types of the eligible studies included

Out of the seventeen eligible studies, 47% (8) were conducted using a quantitative study type, 35% utilised a qualitative approach, and 18% employed mixed-methods analysis. All the included studies focused on the sexual health dimensions of sexual activity, contraceptive use, sexual autonomy, sexual violence and risky sexual behaviour among women with disabilities in SSA (Fig. 3).

### Sexual health focus and countries conducted across sub-Saharan Africa

The results on the sexual health focus show that two studies were conducted on sexual activity [42, 44], ten studies were conducted on contraceptive use [34–40, 45, 48, 49], none was found on sexual autonomy, four studies were conducted on sexual violence [41, 43, 47, 50] whilst one study was conducted on risky sexual behaviour [50] (Table 2).

### Country and sexual health focus mix

**Fig. 4:**
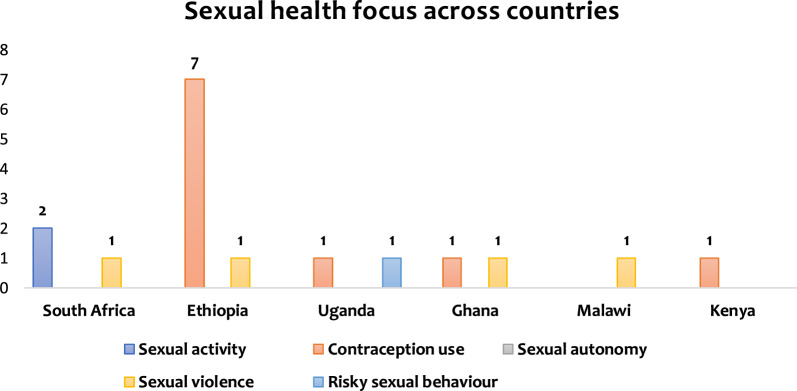
Sexual health focus across the eligible studies countries

Figure 4 shows the country mix and sexual health focus among women with disabilities in SSA. The graph shows that seven studies conducted in Ethiopia were on contraceptive use, whilst one study was conducted on sexual violence. Out of three studies conducted in South Africa, two were on sexual activity, whilst one was on sexual violence; in the same vein, the two studies conducted in Uganda were on contraceptive use and risky sexual behaviour. Ghana had two studies, one on contraceptive use and the other on sexual violence, whilst Malawi and Kenya had one study each, and the studies were on sexual violence and contraceptive use, respectively.

### Main findings from the sexual health dimensions included in the study.

#### Sexual activity

The findings on the eligible included studies suggest that disability stigma may hinder the attainment of normative womanhood and sexual relationships among women with disabilities in South Africa [44], while the persistent gap in access to prevention and treatment for people with disabilities highlights sexual abuse and exploitation as substantial threats to HIV/AIDS prevention efforts within this population [42].

#### Contraceptive use

In Ethiopia, the unmet need for family planning among women with disabilities stands at 24.3% [34]. Only 18% of women have ever used modern contraceptives, with a prevalence rate of 20.2% among currently married women [35]. Family planning utilisation among reproductive-age women with disabilities was 33.7% [36], and among women aged 35 and above, the rate was 27.2%, with injectable contraceptives being the most commonly used method (93.2%) [37]. High awareness of family planning methods was noted at 97.2%, but comprehensive knowledge was low at 32.5% in Ethiopia and Ghana [38, 48]. In rural Ethiopia, the overall unmet need for family planning was 24.08%, with 14.79% for spacing and 9.29% for limiting purposes [39]. In Uganda, 26.1% of women have utilised contraception, with increased uptake linked to access to health facilities and family planning information broadcast on the radio [45]. In Kajiado County, Kenya, 32.0% of women with disabilities use family planning, and 61.2% have negative attitudes towards it, which significantly affects utilisation rates [49]. Among reproductive-age women with disabilities in Ethiopia, 27.3% were current contraceptive users, with 48.5% preferring implants [40].

Several factors influence contraceptive use among women with disabilities. These include age, education level, desired number of children, employment status, and marital status. Attitudes towards family planning also play a significant role, particularly in Kenya, where negative perceptions were prevalent [49]. Access to health facilities and information dissemination, such as radio broadcasts in Uganda, was also crucial for increasing contraceptive uptake [45].

A qualitative response from one of the respondents in a study conducted by Nketsia et al. [48] stated, “*I wish healthcare workers were more patient. I often experience misunderstandings when discussing modern contraceptive issues because they don't know sign language, which is our main form of communication”.*

#### Sexual violence

In South Africa, learners with intellectual disabilities face school-based sexual violence, with frequent occurrences of inappropriate touching, threats, and intimidation [43]. In Ghana, women with disabilities experience a high prevalence of sexual violence, influenced by factors such as poverty, family rejection, isolation, and unemployment, which lead to unwanted pregnancies, divorce, rejection, and psychological trauma [47]. In Malawi, although none of the informants reported childhood sexual abuse, many women described experiencing sexual abuse in adulthood, often involving men who pretended to want to "marry" them but abandoned them once they became pregnant [50]. In Ethiopia, a notably high prevalence of sexual violence was reported among reproductive-age females with disabilities, at 59.8% [41].

Several factors contribute to the risk of sexual violence among women with disabilities. Demographic factors such as urban residence and specific age groups (25–34 and 35–49 years old) increase vulnerability, as seen in Ethiopia [41]. A lack of sexual information and specific disabilities, such as hearing impairments, further elevate the risk. In Malawi, societal attitudes and deceptive behaviours by men exacerbate the issue, leading to psychological trauma and social stigma for the affected women [50].

#### Risky sexual behaviour and sexual autonomy

The main key finding highlights was that risky sexual behaviour was prevalent among women with disabilities and often associated with HIV/AIDS, unwanted pregnancies due to sexual encounters with strangers [46]. Only a study was identified on sexual behaviour whilst no study was found for sexual autonomy based on this study’s selection criteria.

## Discussion

To the best of our knowledge, this is the first scoping review conducted with the aim of understanding the research gap in improving the sexual health outcomes of women with disabilities in SSA with a focus on the following key dimensions of sexual health such as sexual activity, contraceptive use, sexual autonomy, sexual violence and risky sexual behaviour. This current scoping review provides a comprehensive summary of existing research and identifies areas that require further research in improving the sexual health of this population within SSA.

The distribution of previous research conducted across the included five dimensions of sexual health appears to be scanty among women with disabilities in SSA except for contraceptive use, in which case was majorly conducted in Ethiopia [34–40]. The review was unable to map any study on the sexual autonomy of women with disabilities in SSA, whilst few studies were conducted on sexual activity and risky sexual behaviour [42, 44, 46].

For sexual activity among women with disabilities in SSA, this current review found that disability stigma threatens normative womanhood and also that there were higher risk of sexual exploitations [44]. These findings align with a study conducted by Hubbard et al. [51], which concluded that there were higher sexual exploitations among women who were vulnerable compared to those who were not. This finding highlights the significant need for sex education programmes specifically targeting women with disabilities in SSA. These programmes should be customised for this group, as studies have shown that tailored sexual education empowers women with disabilities to make informed decisions, including choices about sexual activity [52, 53].

Regarding contraceptive use, the findings of this review show an unmet need for contraception among women with disabilities in SSA. These findings align with other studies conducted outside Africa regions, which ascertained that family planning services were less accessible to women with disabilities [54, 55]. The possible reasons for this may be due to lack of awareness or knowledge-gap and also limited access to disability-friendly services. The implication of this could result in women with disabilities facing increased chances of experiencing unwanted pregnancies and related adverse reproductive health outcomes [22]. Therefore, to ensure that the contraceptive needs of women with disabilities in SSA are met, it is crucial to draw the attention of key stakeholders and policymakers to the creation of awareness programmes targeting this population [56].

Furthermore, it is essential to provide accurate and timely information and to ensure that contraceptive services are accessible and disability-friendly. This approach aligns with the United Nations' principle of 'leaving no one behind' by promoting inclusivity [57–59]. If the use of contraceptives is low or not improved among this group, this may have a serious impact on the sexual and reproductive health of these women, which could lead to poor health outcomes [56].

The findings on sexual violence in this review revealed that women with disabilities were at risk of sexual violence in SSA. This is also in line with emerging studies that have documented that people with disabilities were at an increased risk of sexual violence [60, 61]. In the same vein, findings have also confirmed that women with a disability were at greater risk of experiencing rape than women without a disability [62, 63]. It has also been noted that suboptimal empowerment could also lead women with a disability to be more likely than women without a disability to report experiencing rape and sexual coercion, among other sexual abuses [64, 65]. Therefore, by understanding that women with disabilities are at greater risk of experiencing sexual violence, there is a need to take deliberate actions to stop the violence through targeted empowerment activities for this key population. In the same vein, there is a dire need to ensure self-protection skills such as consent to sex or sexual autonomy and also to develop a conducive environment and various channels for reporting sexual violence [64, 65].

Furthermore, the present study reveals a high prevalence of risky sexual behaviour among women with disabilities, correlating significantly with HIV/AIDS, unwanted pregnancies due to sexual encounters with strangers [46]. These findings align with previous findings on people with disabilities, which reported an elevated level of sexual risk-taking and its adverse sexual health outcomes [66]. It has been noted that this specific population group face significant barriers to sexual health education and services, which could mitigate these risks and improve overall sexual health outcomes [11].

Lastly, this study's findings were unable to map any study on sexual autonomy among women with disabilities in SSA based on the study’s inclusion and exclusion criteria. However, results from the studies conducted in other regions have indicated little or no sexual autonomy among women with disabilities, with emphasis on being surveilled or controlled by either their partner or family members to be involved in sexual acts [9, 10].

In general, the results of this review indicate the need for designing well-informed educational programmes that are need-based, which could influence behavioural change communication strategies to promote contraceptive use and sexual autonomy and prevent sexual violence and risky sexual behaviour among women with disabilities in SSA. The results reported in this study need to be taken seriously to improve the sexual health of women with disabilities and avert the consequence of adverse health outcomes [11, 17, 56, 57].

## Limitations of the study

Our scoping review on improving sexual health outcomes among women with disabilities in SSA faces several limitations. Firstly, we excluded non-English language articles and grey literature, potentially missing valuable research and introducing bias. Secondly, the narrative synthesis approach was without meta-analysis, which prevented us from providing pooled prevalence rates of the sexual health dimensions considered in this study, affecting the precision of our conclusions. Also, the study heterogeneity in definitions, measurements, and populations may complicate comparisons and generalisability. Finally, potential publication bias and missing data may skew our findings. Future studies should aim to address and overcome these limitations.

## Implications for research and policy

Our findings indicate a significant need for sexual empowerment programmes and targeted interventions to improve access to contraceptive services, increase sexual autonomy and prevent sexual violence and risky sexual behaviour among women with disabilities in SSA. Policies should ensure the provision of disability-friendly sexual health services and educational programmes tailored to women with disabilities are prioritised in SSA. Additionally, stakeholders and policymakers must ensure timely awareness campaigns and support systems for these women to enhance their sexual health and uphold the principle of inclusivity by ensuring that healthcare systems adapt to offer accessible and inclusive sexual health services, ensuring women with disabilities can effectively communicate their needs. This could close the adverse sexual health outcomes gaps among this population through well-informed policies and evidence-based strategies that are crucial to improving the sexual and reproductive health outcomes for women with disabilities in SSA.

## Conclusion and recommendations for future research

This review showed that there were few or scarce studies on sexual activity, contraceptive use, sexual autonomy, sexual violence and risky sexual behaviour among women with disabilities in SSA and even where the studies were substantial (contraceptive use), the majority of the studies were conducted in Ethiopia. Furthermore, this current review shows that women with disabilities were less likely to use contraceptives and were at risk of sexual violence. Therefore, there is a need to pool all relevant stakeholders from the government, communities, and development partners to deliberately coin policies and programmes that empower access to contraceptives and prevent sexual violence among women with disabilities. Moreover, further studies should be conducted to explore the needs of women with disabilities regarding contraceptive use and also their perspectives regarding preventing and reporting sexual violence. In the same vein, future studies should consider examining other dimensions of sexual health, such as sexual autonomy, sexual activity and risky sexual behaviour of women with disabilities that were not available or were scarce in the literature.

### Supplementary Information

Supplementary Material 1.

## Data Availability

All data generated and analysed during this study are included in this manuscript as supplementary information.

## Abbreviations

SSA: Sub-Saharan Africa
PRISMA: Preferred Reporting Items for Systematic Reviews and Meta-Analyses
PROSPERO: International Prospective Register of Systematic Reviews
MeSH: Medical Subject Headings
ICPD: International Conference on Population and Development
SRHR: Sexual and reproductive health and rights
MESH: Medical Subject Headings
