# Supplementary material for: Improving the sexual health of women with disabilities in sub-Saharan Africa: a scoping review of published studies
Source: Reprod Health. 2024 Aug 6;21:116. doi: 10.1186/s12978-024-01859-1 (PMC11304614; doi:10.1186/s12978-024-01859-1)
Supplement: Supplementary file 1 — Supplementary Material 1. [file 12978_2024_1859_MOESM1_ESM.docx]

**Supplementary Appendix II – Search keywords development**

**Keywords used**

- Sexual Health
- Sexual Behavior
- Sexual Activity
- Risky Sexual Behavior
- Sexual Violence
- Sexual Autonomy
- Contraceptive Use
- Disabled Persons
- Women with Disabilities
- Sub-Saharan Africa
- Africa South of the Sahara
- Health Services Accessibility
- Empowerment
- Health Education
- HIV Infections
- Unplanned Pregnancy
- Family Planning Services
- Sexual Empowerment
- Sexual Rights
- Sexual Risk-Taking

**Previous research question**

How do the dimensions of sexual activity, contraceptive use, sexual autonomy, sexual violence, and risky sexual behavior impact the sexual health outcomes of women with disabilities in sub-Saharan Africa?

**Current research question**

What is the volume and coverage of current studies on improving sexual health including sexual activity, contraceptive use, sexual autonomy, sexual violence, and risky sexual behavior of women with disabilities in sub-Sharan Africa?

**Supplementary Appendix 1 - Search process**

1. Search strings - sexual activity of women with disability in sub-Saharan Africa - 249

("sexual actvity"[MeSH Terms] OR ("sexual"[All Fields] AND " actvity"[All Fields]) OR "sexual actvie"[All Fields]) AND ("womans"[All Fields] OR "women"[MeSH Terms] OR "women"[All Fields] OR "woman"[All Fields] OR "women s"[All Fields] OR "womens"[All Fields]) AND ("disabilities"[All Fields] OR "disability"[All Fields] OR "disabled persons"[MeSH Terms] OR ("disabled"[All Fields] AND "persons"[All Fields]) OR "disabled persons"[All Fields] OR "disabled"[All Fields] OR "disablement"[All Fields] OR "disablements"[All Fields] OR "disabling"[All Fields] OR "disablity"[All Fields]) AND ("africa south of the sahara"[MeSH Terms] OR ("africa"[All Fields] AND "south"[All Fields] AND "sahara"[All Fields]) OR "africa south of the sahara"[All Fields] OR ("sub"[All Fields] AND "saharan"[All Fields] AND "africa"[All Fields]) OR "sub saharan africa"[All Fields])

1. Search strings – contraceptive use among women with disability in sub-Saharan Africa - 244

("contraceptive"[MeSH Terms] OR ("sexual"[All Fields] AND "behavior"[All Fields]) OR " contraception"[All Fields]) AND ("womans"[All Fields] OR "women"[MeSH Terms] OR "women"[All Fields] OR "woman"[All Fields] OR "women s"[All Fields] OR "womens"[All Fields]) AND ("disabilities"[All Fields] OR "disability"[All Fields] OR "disabled persons"[MeSH Terms] OR ("disabled"[All Fields] AND "persons"[All Fields]) OR "disabled persons"[All Fields] OR "disabled"[All Fields] OR "disablement"[All Fields] OR "disablements"[All Fields] OR "disabling"[All Fields] OR "disablity"[All Fields]) AND ("africa south of the sahara"[MeSH Terms] OR ("africa"[All Fields] AND "south"[All Fields] AND "sahara"[All Fields]) OR "africa south of the sahara"[All Fields] OR ("sub"[All Fields] AND "saharan"[All Fields] AND "africa"[All Fields]) OR "sub saharan africa"[All Fields])

1. Search strings - sexual autonomy of women with disability in ** - 335

("sexual autonomy"[MeSH Terms] OR ("sexual"[All Fields] AND " autonomy"[All Fields]) OR "sexual independence"[All Fields]) AND ("womans"[All Fields] OR "women"[MeSH Terms] OR "women"[All Fields] OR "woman"[All Fields] OR "women s"[All Fields] OR "womens"[All Fields]) AND ("disabilities"[All Fields] OR "disability"[All Fields] OR "disabled persons"[MeSH Terms] OR ("disabled"[All Fields] AND "persons"[All Fields]) OR "disabled persons"[All Fields] OR "disabled"[All Fields] OR "disablement"[All Fields] OR "disablements"[All Fields] OR "disabling"[All Fields] OR "disablity"[All Fields])

1. Search strings - sexual violence of women with disability in Africa - 247

("sexual violence"[MeSH Terms] OR ("sexual"[All Fields] AND " violence"[All Fields]) OR "sexual violent"[All Fields]) AND ("womans"[All Fields] OR "women"[MeSH Terms] OR "women"[All Fields] OR "woman"[All Fields] OR "women s"[All Fields] OR "womens"[All Fields]) AND ("disabilities"[All Fields] OR "disability"[All Fields] OR "disabled persons"[MeSH Terms] OR ("disabled"[All Fields] AND "persons"[All Fields]) OR "disabled persons"[All Fields] OR "disabled"[All Fields] OR "disablement"[All Fields] OR "disablements"[All Fields] OR "disabling"[All Fields] OR "disablity"[All Fields]) AND ("africa"[MeSH Terms] OR "africa"[All Fields] OR "africa s"[All Fields] OR "africas"[All Fields])

1. Search strings – risky sexual behaviour among women with disability in Africa - 287

("risky sexual behaviour"[MeSH Terms] OR ("risky sexual"[All Fields] AND " risky behaviour"[All Fields]) OR " risky sexual behavi**"[All Fields]) AND ("womans"[All Fields] OR "women"[MeSH Terms] OR "women"[All Fields] OR "woman"[All Fields] OR "women s"[All Fields] OR "womens"[All Fields]) AND ("disabilities"[All Fields] OR "disability"[All Fields] OR "disabled persons"[MeSH Terms] OR ("disabled"[All Fields] AND "persons"[All Fields]) OR "disabled persons"[All Fields] OR "disabled"[All Fields] OR "disablement"[All Fields] OR "disablements"[All Fields] OR "disabling"[All Fields] OR "disablity"[All Fields]) AND ("africa"[MeSH Terms] OR "africa"[All Fields] OR "africa s"[All Fields] OR "africas"[All Fields])
